# Differential microbial responses to antibiotic treatments by insecticide-resistant and susceptible cockroach strains (*Blattella germanica* L.)

**DOI:** 10.1038/s41598-021-03695-w

**Published:** 2021-12-17

**Authors:** Zachery M. Wolfe, Michael E. Scharf

**Affiliations:** Department of Entomology, Purdue University, West Lafayette, IN 47907 USA

**Keywords:** Microbial ecology, Microbiome

## Abstract

The German cockroach (*Blattella germanica* L.) is a major urban pest worldwide and is known for its ability to resist insecticides. Past research has shown that gut bacteria in other insects can metabolize xenobiotics, allowing the host to develop resistance. The research presented here determined differences in gut microbial composition between insecticide-resistant and susceptible German cockroaches and compared microbiome changes with antibiotic treatment. Cockroaches received either control diet or diet plus kanamycin (KAN) to quantify shifts in microbial composition. Additionally, both resistant and susceptible strains were challenged with diets containing the insecticides abamectin and fipronil in the presence and absence of antibiotic. In both strains, KAN treatment reduced feeding, leading to higher doses of abamectin and fipronil being tolerated. However, LC50 resistance ratios between resistant and susceptible strains decreased by half with KAN treatment, suggesting gut bacteria mediate resistance. Next, whole guts were isolated, bacterial DNA extracted, and 16S MiSeq was performed. Unlike most bacterial taxa, *Stenotrophomonas* increased in abundance in only the kanamycin-treated resistant strain and was the most indicative genus in classifying between control and kanamycin-treated cockroach guts. These findings provide unique insights into how the gut microbiome responds to stress and disturbance, and important new insights into microbiome-mediated insecticide resistance.

## Introduction

Insecticide-resistant strains of insect pests are more prevalent than ever before. Genetic mutations in insect species allow some insects to resist high concentrations of insecticide^1,2^. Once these insects reproduce, their offspring also contain the mutations that code for resistance. If the same insecticide is consistently applied to the same insect population in the same geographic location, within a small number of generations the majority of the population will express genetic resistance to the specific insecticide. This cycle significantly reduces the lethality of insecticides with each subsequent generation of insecticide-resistant pests. In the United States alone, total pesticide resistance accounts for $1.5 billion in total economic losses each year^3^. It is essential to determine the causes of insecticide resistance to extend the effective useful life of active ingredients and prevent the spread of dangerous, damaging and undesirable insects.

Insecticide resistance is characterized as either a behavioral or a physiological adaptation of an insect species to a respective toxicant. Behavioral insecticide resistance can be characterized by a change in the actions or responses of an insect in the presence of the insecticide or its formulation components. For example, cockroach strains that were once attracted to a particular glucose sweetener of a bait matrix now find the ingredient unpalatable; the mutant strains no longer consume the bait and the bait matrix becomes ineffective for pest control^4,5^. Physiological resistance, on the other hand, describes a change in the biochemical composition or microbiome of an insect species. For example, a species may overproduce endogenous detoxification enzymes in the presence of an insecticide. Insect physiology typically influences insect behavior, and likewise insect behavior leads the way to the development of unique physiological traits^6^.

The German cockroach (*Blattella germanica*) is an invasive pest species that has infested houses, apartments, hospitals, schools, and other urban facilities on a worldwide scale^7–9^. German cockroaches are widespread in many urban areas, particularly in low-income apartments and housing communities^9,10^. German cockroaches pose a hazard to human health and well-being by carrying pathogens and pathogenic organisms, instigating allergic reactions and scattering fecal matter and carcasses throughout residences^7,11^. Although it prefers foods rich in carbohydrate compared to foods rich in fat and protein content^12^, the German cockroach will eat virtually any type of food substance it encounters^13^, allowing it to adapt easily to unkempt areas such as kitchens, bathrooms and pantries. Additionally, German cockroaches forage at random and cannot detect food or water more than a few centimeters away^14^, forcing German cockroach colonies to spread out and colonize new areas quickly.

*Blattella germanica* is highly adaptive to its environment due to its extremely generalist feeding behavior and its ability to withstand nutritional imbalances^15^. German cockroach populations can persist in severely toxic surroundings over time thanks in part to point mutations in their genome. For example, the German cockroach has previously shown physiological knockdown-resistance to pyrethroid insecticides with a single mutation in its voltage-gated sodium channel^16^. The German cockroach has also developed resistance to cyclodiene insecticides, which act by antagonizing GABA action on the GABA receptors in insects^17^. Through a mutational change in the biochemical properties of the target site of the GABA receptor itself, the affinity of the receptor to bind with cyclodienes is reduced significantly, which gives cockroaches up to 100-fold resistance to cyclodiene insecticides^17^.

Research using *Spodoptera frugiperda* has shown that some gut bacteria in insect species break down xenobiotics and toxic compounds, facilitating and enhancing an insect’s ability to resist insecticidal compounds^18^. The coffee berry borer (*Hypothenemus hampei*), a devastating pest to coffee plantations across the world, has gut microbes that have developed the ability to degrade the insecticidal compound caffeine^19^. The apple maggot (*Rhagoletis pomonella*) contains a symbiotic bacterium (*Pseudomonas melophthora*) which can degrade up to six different insecticides that would otherwise control the apple maggot^20^.

Since the German cockroach is an insect species notorious for its ability to tolerate insecticide applications and is also known to host a plethora of microbial gut symbionts^21–24^, there is reason to suspect that these gut microbes have an impact on insecticide resistance, tolerance, and/or degradation. Isolating these microbial species and studying how they react to insecticidal compounds is crucial to determine the mechanisms of insecticide resistance in the German cockroach and in its microbial symbionts. Learning which bacterial symbionts are present in insecticide-resistant and susceptible cockroaches will give us clues as to which bacterial symbionts might help degrade and detoxify insecticides.

Dysbiosis is broadly defined as deleterious compositional and functional alterations of the gut microbiome, many of which are thought to contribute to a range of conditions of ill health^25^. Thanks in part to the decreasing cost of next-generation sequencing, this field of research has expanded exponentially in the past decade as medical researchers race to find treatments and cures for a myriad of gastrointestinal disorders like Crohn’s disease and irritable bowel syndrome. Dysbiosis in arthropods, however, remains largely unexplored. Investigating the gut microbiome of pest insects would allow insecticide manufacturers to develop dysbiosis-based synergists to increase the effectiveness of other active ingredients. This method would be particularly effective for pest insects which orally feed on bait matrices, such as German cockroaches.

Recent research in German cockroaches has revealed how insecticide resistance can affect gut microbial composition and stability, along with the physiology and life history of the host. Zhang et al*.*^26^ observed that beta-cypermethrin-resistant cockroaches exhibited a delayed development period and reduced adult longevity compared with susceptible cockroaches—most importantly, these researchers concluded that variation in gut microbiota, especially those related to growth and development, was an important influencing factor when comparing resistant and susceptible cockroaches. While this research does not directly relate gut microbiota to insecticide metabolism, it is a key study indicating that host fitness costs and physiology can be affected and reflected by the gut microbiome and the species present within.

Additional studies have recorded the impact of antibiotics on gut microbial communities in German cockroaches. Rosas et al*.*^27^ applied rifampicin to German cockroach populations which exerted a drastic effect on gut microbiota composition, although composition recovered in the second generation in the case where antibiotic was not added to the diet. The endosymbiotic *Blattabacterium* population, exclusively found in cockroach fat bodies, remained unaffected by the antibiotic treatment of adults during the first generation but was strongly reduced in the second generation, suggesting that *Blattabacterium* is sensitive to rifampicin only during the infection of mature oocytes, when it is in an extracellular stage. This theme of gut microbial alteration and subsequent reversion was corroborated by two 2020 studies, Dominguez-Santos et al*.*^28^ and Li et al*.*^29^. Dominguez-Santos et al*.* found that in an untreated second-generation population that comes from an antibiotic-treated first-generation, the microbiota is not yet stabilized at nymphal stages. However, once feces of a control population were added to the diet, microbiota had fully recovered by the time the second-generation reached adulthood. Li et al*.* treated German cockroach with the antibiotics levofloxacin and gentamicin and found that within 14 days of discontinuing antibiotic treatment, the number of culturable gut bacteria returned to its original level (pre-antibiotic). However, the composition of the new bacterial community with greater abundance of antibiotic-resistant bacteria was significantly different from the original community.

The objective of this research was to compare the whole gut bacterial profiles of insecticide resistant and susceptible *B. germanica* and determine how these profiles, as well as the structure and function of the gut microbiome, change in the presence of an antibiotic. In parallel, we also investigated oral toxicity of the two insecticide bait active ingredients abamectin and fipronil in resistant and susceptible cockroach strains, with and without antibiotic treatment. We hypothesized that there would be differences in gut microbial structure and function between insecticide resistant and susceptible cockroach strains as well as differences in gut microbial structure and function between antibiotic and control-treated cockroaches. Our findings show antibiotic-induced dysbiosis in only the resistant strain, as well as possible roles for gut microbiota in insecticide resistance and in facilitating insecticide toxicity under basal conditions.

## Results

### Insecticide bioassays and antibiotic synergism

**Figure 1:**
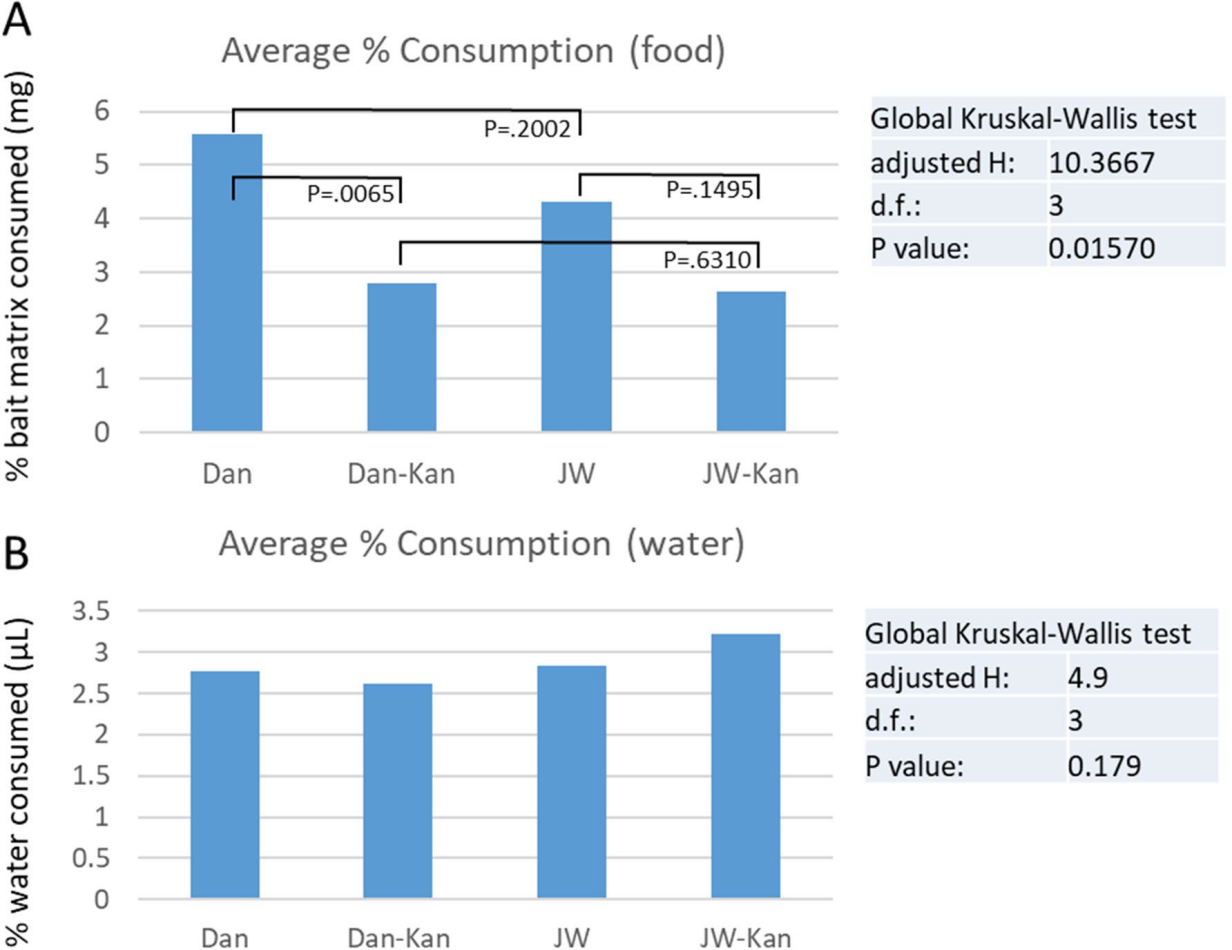
(**A**) represents the average percent of bait matrix consumed per cockroach, per treatment type, per strain. Danville (R) cockroaches consumed significantly less bait when treated with kanamycin, meanwhile J-wax (S) cockroaches did not consume significantly different quantities of bait. (**B**) represents the average percent of liquid (NanoPure water or kanamycin-infused NanoPure water) consumed per cockroach, per treatment type, per strain. There were not significant differences in liquid consumed and/or evaporated between all treatment types.

Probit calculations followed Finney^30^. Under basal conditions, the Danville resistant (R) strain showed significant resistance to both abamectin and fipronil upon ingestion, with LC50 resistance ratios relative to the susceptible J-wax (S) strain being 4.844 and 7.882, respectively (Table 1). In both strains and with both insecticides, KAN treatment led to higher doses being required to cause median mortality. However, resistance ratios between the resistant and susceptible strains decreased by approximately half with KAN treatment, suggesting potential roles for gut bacteria in mediating resistance. Parallel investigations into feeding effects of KAN treatment revealed that food consumption decreases with KAN treatment, but feeding amounts were identical between R and S strains (Fig. 1). Thus, the decrease in resistance ratios after KAN treatment suggest a significant influence of gut microbiome on resistance.

**Table 1:** Probit analysis of bioassay results after 72 h of abamectin and fipronil treatments.

Insecticide	Strain	KAN	N	Slope	ChiSq-test (χ^2^) Sig	LC50 (μg/dish)	95% CI (lower)	95% CI (upper)	Kan ± ratio	Resistance ratio
Abamectin	Jwax (S)	+	429	0.839 ± 0.180	0.99	5.191	2.309	11.672	2.800	
		−	424	1.25 ± 0.127	0.89	1.854	1.045	3.288		
	Danville (R)	+	432	1.50 ± 0.121	0.00	10.951	6.334	18.933	1.219	2.109
		−	429	1.88 ± 0.102	0.00	8.981	5.657	14.258		4.844
Fipronil	Jwax (S)	+	465	1.02 ± 0.152	0.00	0.180	0.091	0.359	7.911	
		−	469	1.46 ± 0.106	0.06	0.023	0.014	0.037		
	Danville (R)	+	463	0.888 ± 0.203	0.00	0.680	0.272	1.701	3.781	3.767
		−	473	1.24 ± 0.133	0.00	0.180	0.099	0.327		7.882

Separated by strain and kanamycin exposure (sample size = 10). Kan ± Ratio = LC50 of KAN-treated/LC50 of untreated. Resistance Ratio = LC50 of Danville (R)/LC50 of J-wax (S). Chi-squared values are within acceptable range for conducting probit.

### 16S sequencing: alpha diversity

**Figure 2:**
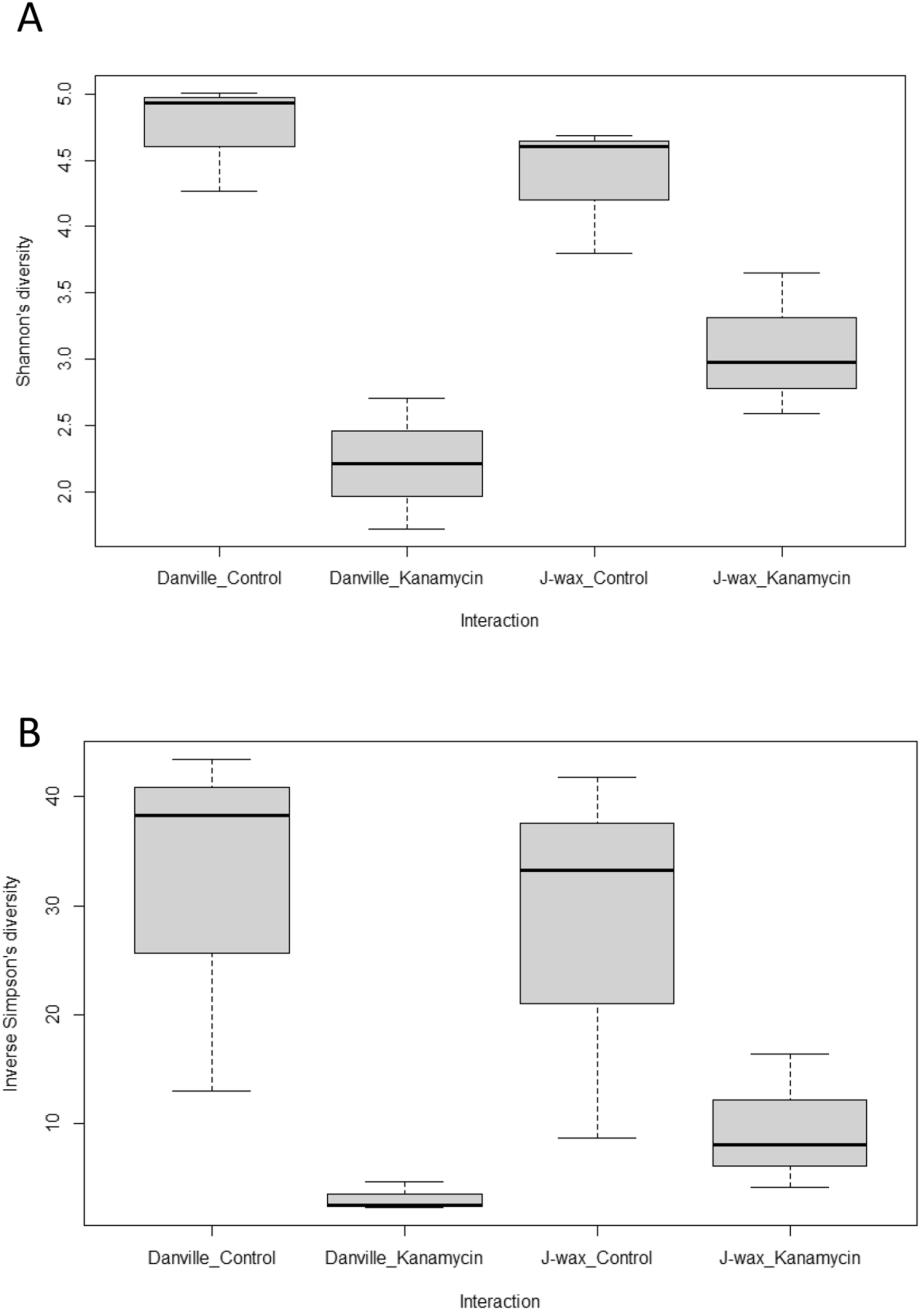
Boxplots showing the median (horizontal line in the box), interquartile range (IQR, the box), minimum and maximum (lines below and above the box, respectively) of alpha diversity (top: Shannon (**A**), bottom: Inverse Simpson (**B**)) categorized by treatment. P-values for global Kruskal–Wallis comparisons between strain and treatment combinations are 0.02607 (Shannon) and 0.05222 (inverse Simpson).

Antibiotic treatment had a significant effect on microbial diversity in both Danville (R) and J-wax (S) guts (p-values: Shannon: 0.000135, inverse Simpson: 0.0107). However, there were not significant differences in gut microbial diversity between the Danville (R) and J-wax (S) cockroach strains to the genus level when KAN treatment was not considered (p-values: Shannon: 0.411204, inverse Simpson: 0.8528). The p-values for combined Treatment:Strain interaction were 0.058173 and 0.5006 for Shannon and inverse Simpson’s diversity, respectively. Alpha diversity metrics extend just beyond the P < 0.05 statistical significance threshold, however differences in diversity can still be observed (Fig. 2).

### 16S sequencing: beta diversity

**Figure 3:**
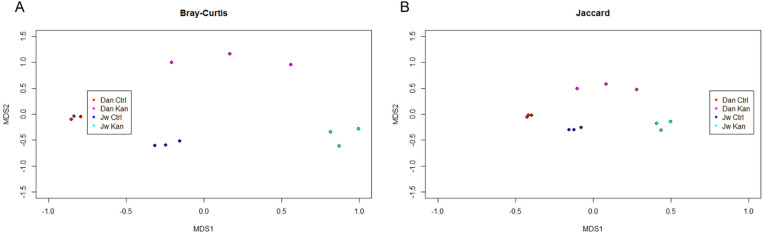
NMDS of beta-diversity (left: Bray–Curtis (**A**), right: Jaccard (**B**)) categorized by treatment and strain combined. Overall significance of the models as determined by PERMANOVA: Bray–Curtis Shannon (P = 0.000999), inverse Simpson (P = 0.001998), Jaccard Shannon (P = 0.000999), inverse Simpson (P = 0.000999).

Bacterial communities were unique to each treatment type in terms of their taxonomic diversity (Fig. 3). Kanamycin-treated samples were clustered less densely compared to their control counterparts, indicating the kanamycin treatment had slightly unique and different effects on each sample.

### Differential bacterial abundance by treatment and strain

**Figure 4:**
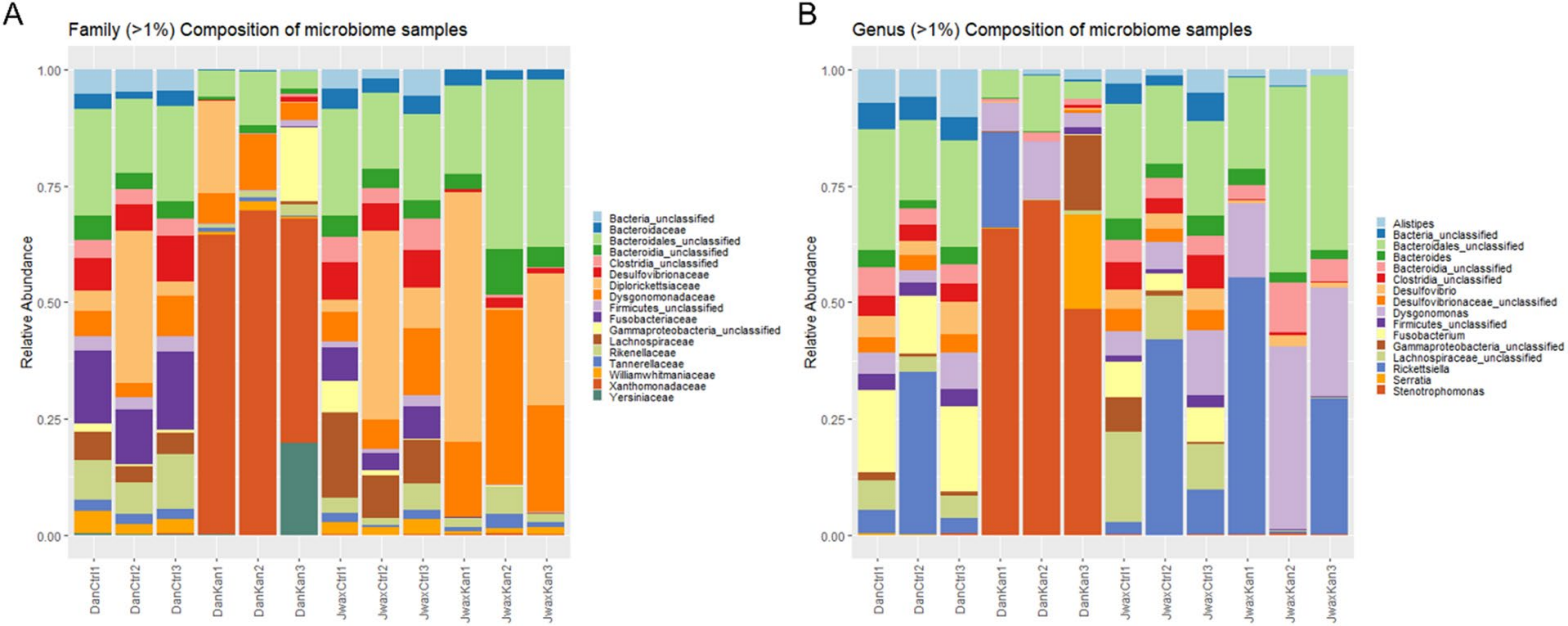
Relative abundance of families (**A**) and genera (**B**) with over 1% composition throughout the entire sequence categorized by treatment and replication. Bars are colored by family (**A**) and genus (**B**). Group is categorized by strain, treatment and replication (sample size = 10).

**Figure 5:**
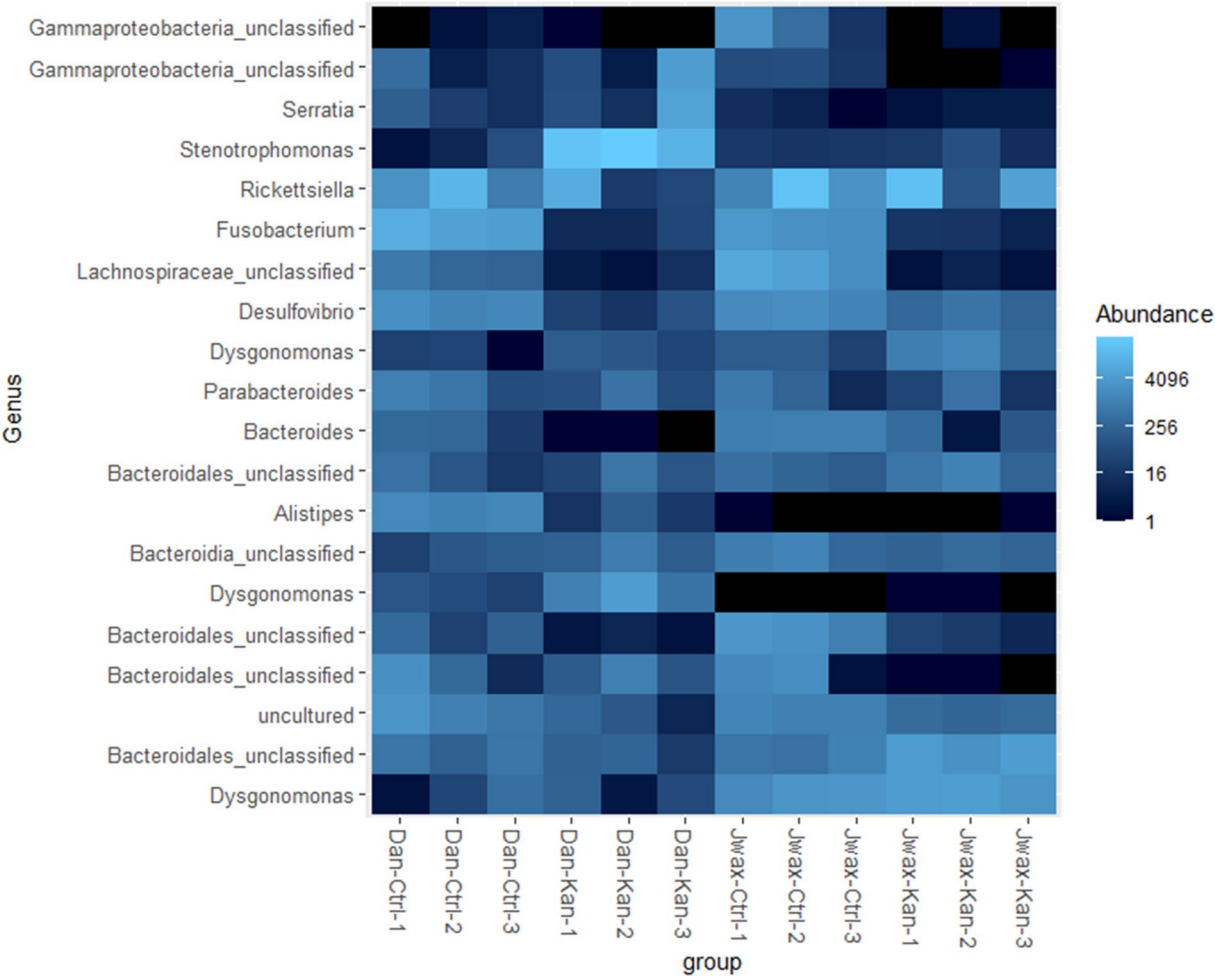
Heat map of top 20 genera throughout the entire 16S MiSeq. Figure includes uncultured and unclassified bacterial genera. Group is categorized by strain, treatment and replication. Black = absent.

When the Danville strain was fed antibiotics, *Stenotrophomonas* spp. was substantially greater in relative abundance than all other genera combined (Fig. 4). In addition to an increase in *Stenotrophomonas*, kanamycin exposure effectively decreased the relative quantities of all other bacterial genera except for *Dysgonomonas*, *Alistipes* and a select group of unclassified *Bacteriodales* spp. While relative quantities of each genus might vary by treatment type and even by replication within the same treatment type, most taxa were retained between each strain (Fig. 5).

### Differential bacterial abundance using DESeq2

**Figure 6:**
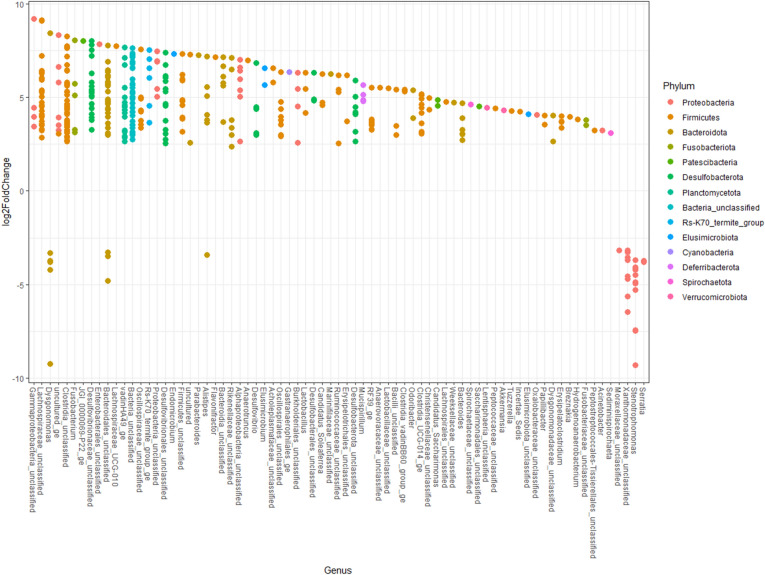
Differential abundance using DESeq between treatment types (control vs kanamycin). Values are colored by phylum and labeled on the x-axis by genus. Positive log2 FoldChange values indicate the presence of a genus is more indicative of a control treatment, whereas negative log2FoldChange values indicate the presence of a genus is more indicative of a kanamycin (antibiotic) treatment.

Figure 6 indicates the differential abundance using DESeq between treatment types (control vs kanamycin) colored by phylum and labeled by genus^31^. A select group of genera belonging to the *Proteobacteria* phylum (including *Stenotrophomonas* spp.), *Dysgonomonas*, *Alistipes* and some unknown *Bacteroidota* taxa increased in relative quantity once the microbiome was exposed to kanamycin. Most other bacterial taxa decreased in relative quantity after kanamycin exposure.

### LEfSe (linear discriminant analysis effect size)

**Figure 7:**
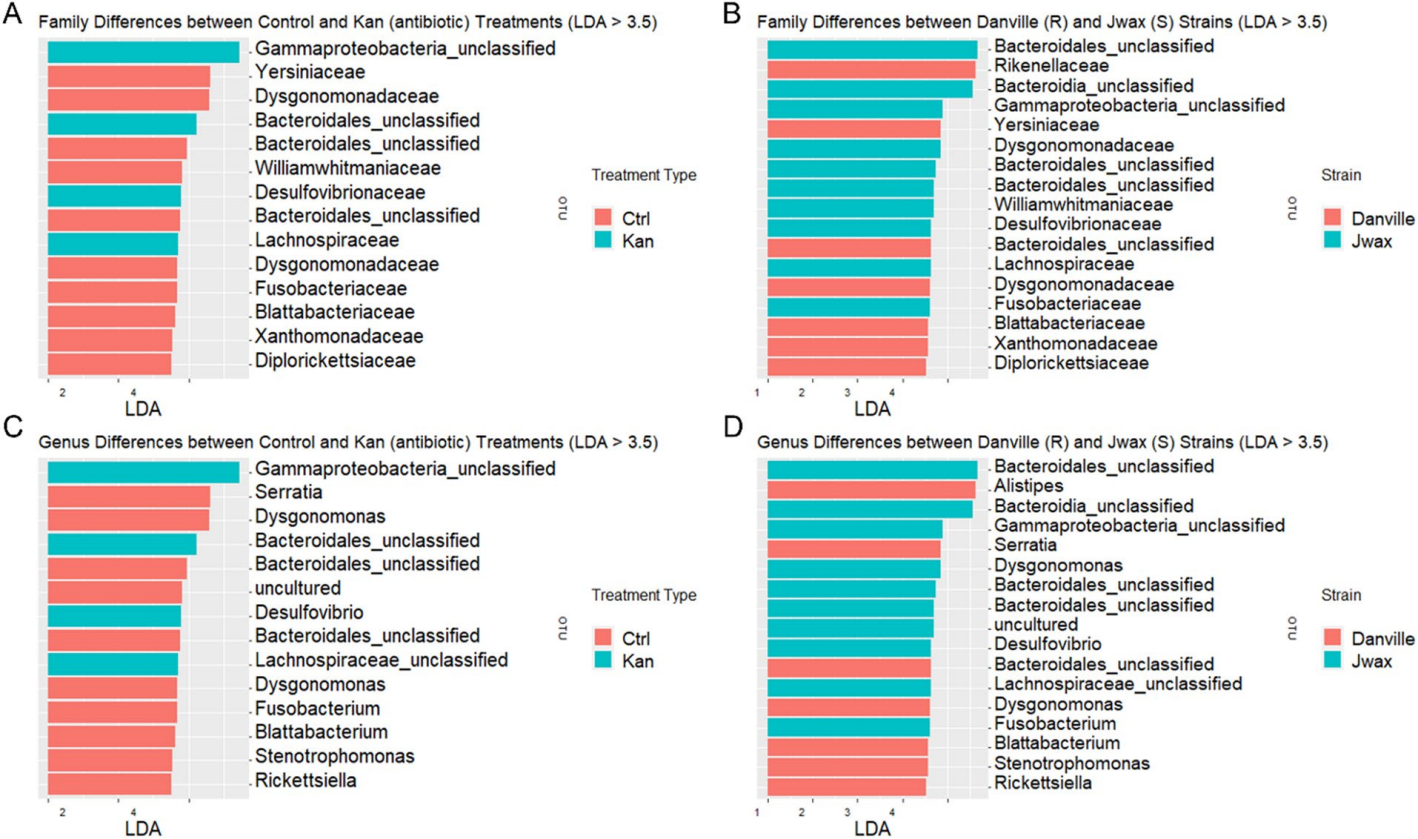
LEfSe (Linear discriminant analysis Effect Size). Differences are measured by Linear Discriminant Analysis (LDA). The graphs represent the families (**A**) and genera (**C**) most likely to differ between treatment types, as well as the families (**B**) and genera (**D**) most likely to differ between strains. *Alistipes* was more likely to be present in Danville (R) roaches compared to J-wax (S) roaches, meanwhile unidentified species from the order *Bacteroidales* and the diverse class *Gammaproteobacteria* were more likely to be present in J-wax (S) roaches.

Significant differences in OTUs between strains and treatment types were identified by LEfSe analysis^32^. LDA scores are shown in Fig. 7A–D. LEfSe analysis confirmed the same taxa as the prior differential abundance analyses. *Alistipes* was more likely to be present in Danville (R) roaches compared to J-wax (S) roaches, meanwhile unidentified species from the order *Bacteroidales* and the very diverse class *Gammaproteobacteria* were more likely to be present in J-wax (S) roaches. The majority of bacteria associated with KAN treatment are previously unidentified or unknown species.

## Discussion

This study investigated microbiome differences between insecticide-resistant and susceptible cockroach strains; specifically, resistance to the bait insecticide active ingredients fipronil and abamectin. We found that pre-treatment with the antimicrobial compound kanamycin (KAN) led to reductions in resistance levels and increased basal toxicity levels in both resistant and susceptible strains tested. 16S bacterial sequence surveys revealed a wide variety of undescribed bacterial taxa, but also both strains were more similar before KAN treatment than after, with a stronger dysbiosis effect in the resistant strain. The discovery of such a wide variety of undescribed bacterial taxa identified in this study is of significant interest; it is possible that these unique bacteria might provide niche benefits to the cockroach host or other gut symbionts, especially in terms of xenobiotic detoxification.

### Insecticide bioassays and implications

The abamectin and fipronil challenges reveal that KAN treatment resulted in higher insecticide tolerance in both the R and S strains tested. However, KAN treatment also decreased resistance ratios by approximately half for both insecticides, suggesting that gut microbiota increase resistance for both abamectin and fipronil. This resistance could be explained by either feeding behavior or by the activation of insecticidal compounds by microbial enzymes—particularly in the case of fipronil, which has two active forms (one being the parent compound itself, and also the sulfone metabolite) which are both toxic to cockroaches^33,34^. There is yet to be a documented case of microbial insecticide activation or detoxification in German cockroach, however, comprehensive research on the gut microbiome of German cockroach has just begun. More studies on host feeding, metabolism and degradation are needed before we can determine specific relationships these microbes might have with their host, or perhaps each other.

### Microbial diversity

Cockroach guts treated with kanamycin were less diverse than cockroach guts in the control group, suggesting that kanamycin eliminated a wide variety of bacterial taxa from the whole gut during the 72-h treatment window before gut extraction. Antibiotic treatment had a significant effect on alpha diversity in both the Danville and J-wax population. The Danville and J-wax cockroach strains do not have significant differences in gut bacterial taxa when treatment is not considered, at least to the genus level, while combined treatment and strain interaction yielded a significance value of 0.058173 and 0.5006 for Shannon and inverse Simpson’s diversity, respectively. Based on significance at the 90% confidence level (which accounts for type II error), the combined effects of treatment and strain were indicative of how microbiota shift in the gut when challenged with an antibiotic.

Our findings suggest the Danville (R) strain has a gut physiology which allows for a unique dysbiosis effect in the presence of kanamycin, while the J-Wax (S) strain’s physiological shift is less pronounced. Whether or not this dysbiosis is related to insecticide resistance at a host population level is yet to be confirmed, but the bacterial taxonomic differences between strains are considerable. Further investigating the metabolomic functions performed by these microbes will help reveal the relationships between bacterial species and the structure and function of the host gut microbiome.

### Abundance and taxa of interest

While the presence of *Dysgonomonas* and *Alistipes* spp. were higher in the Danville strain, they are present to a reduced extent in the guts of J-wax roaches as well. It is possible that there are further differences between the two strains at the species level. *Dysgonomonas* has been previously isolated from the guts of the subterranean termite *Reticulitermes speratus* and researchers suggest this genus requires heme to grow^35,36^. *Dysgonomonas* has not been well-studied, especially outside of human guts, so it would be inappropriate to draw conclusions on insecticide resistance based on its presence in a resistant cockroach strain. *Alistipes* is a nascent sub-branch genus of the *Bacteroidetes* phylum which are commonly associated with chronic intestinal inflammation in humans^37^ and was first discovered in samples of children with appendicitis^38^. *Alistipes* has one of the highest numbers of putrefaction pathways amongst human gut commensal bacteria. Putrefaction is the fermentation of undigested proteins in the GI tract which typically leads to bacterial production of harmful (or occasionally helpful) metabolites^39,40^. Similar to *Dysgonomonas*, the authors cannot presently draw conclusions about the contributions of *Alistipes* spp. in relation to insecticide resistance and degradation, and these genera are likely (but not conclusively) naturally present in different relative abundances between Danville (R) and J-wax (S).

*Stenotrophomonas* spp. are present in every sequence sample to a relative extent, but no more so than in the Danville-Resistant cockroaches that were fed kanamycin. *Stenotrophomonas* is a genus known for its role in the nitrogen and sulfur cycles in the soils of various ecosystems; it has the ability to detoxify xenobiotics and break down complex organic molecules^41^, which might allow a strain of insecticide resistant German cockroaches to tolerate higher doses of insecticides. Also, many *Stenotrophomonas* spp. have a high level of intrinsic resistance to antibiotics^41^ which could also explain why it was able to overwhelmingly colonize the gut microbiome once kanamycin was introduced; kanamycin was clearly less effective at eliminating *Stenotrophomonas* compared to other bacterial genera. Introducing a disturbance (in this case, an antibiotic) to the microbiome most likely allowed for substantially tolerant *Stenotrophomonas* bacteria to take advantage of resources in the gut without competition from other microorganisms. Since *Stenotrophomonas* can effectively decompose organic compounds, perhaps this genus consumed dead or dying bacteria in the gut (a result of kanamycin treatment) and grew in quantity over 72 h as a result. Alternatively, *Stenotrophomonas* could be filling niches leftover from other dead or dying bacteria, explaining the growth after 72 h.

Firmicutes was among the phyla most sensitive to kanamycin exposure. Firmicutes is widely diverse and has been studied in both human and animal gut microbiology, especially in its links to obesity^42,43^. Many of these Firmicutes are in class Clostridia, a common digestive tract bacterium consisting of only anaerobes^44^. Research on the Turkestan cockroach (*Shelfordella lateralis*) suggests that both gut tissue and microbiota contribute to oxygen consumption and suggest that oxygen status in the gut influences microbial colonization success^45^. This same principle could hold true of German cockroach gut microbiota as well; if so, we could expect to see variable microbial alpha or beta diversity metrics based on oxygen consumption or concentration in host tissue. Oxygen consumption was not measured in this experiment, but we recommend follow-up research to determine how oxygen presence (and concentration) might affect the gut microbiome (and coinciding potential insecticide resistance and susceptibility) in other cockroach species.

The family *Lachnospiraceae* (phylum *Firmicutes*, class *Clostridia*) contains anaerobic bacteria that are routinely isolated from the gastrointestinal tract of animals^46^. These bacteria are motile, curved rods, and usually stain Gram negative or weakly Gram positive^46^. *Lachnospiraceae* has mostly been found in mammalian digestive tracts; its main function is to digest complex plant polysaccharides via hydrolysis^47^. Members of *Lachnospiraceae* have been linked to obesity and protection from colon cancer in humans, mainly due to the association of many species with the production of butyric acid, a substance that is important for both microbial and host epithelial cell growth^48^. *Lachnospiraceae* likely did not play a role in insecticide degradation in this experiment, although more studies should be implemented to determine how this family might degrade a pro-insecticide prone to hydrolysis (i.e., indoxacarb).

*Blattabacterium* spp present in the sequence survey likely came from fat bodies outside of the digestive tract^49,50^ and thus it is a possible contaminant to our whole gut sample. For this reason, *Blattabacterium* spp. were eliminated from downstream diversity analyses.

### Comparison to previous studies

Pérez-Cobas et al*.*^51^ pyrosequenced the hypervariable regions V1–V3 of the 16S rRNA gene of the whole bacterial community of German cockroach when exposed to different diets. Three diets differing in protein were tested at two time points in lab-reared individuals. In addition, the gut microbiota of wild adult cockroaches was also analyzed. The most abundant families sequenced were *Porphyromonadaceae* (Bacteroidetes), *Ruminococcaceae* (Firmicutes), *Rikenellaceae* (Bacteroidetes), *Lachnospiraceae* (Firmicutes), *Desulfovibrionaceae* (Proteobacteria) and *Bacteroidaceae* (Bacteroidetes)^34^.

Pietri et al*.*^23^ investigated whole guts from untreated German cockroaches, or cockroaches continuously exposed to 0.5% doxycycline (another antibiotic) for 4 days before dissecting guts and surveying bacterial 16S rRNA genes. Sequence results showed taxa consisting primarily of *Proteobacteria*, *Bacteroidia*, *Firmicutes* and *Fusobacteria*^23^. These researchers also successfully demonstrated that gut microbiota can differ between insecticide-resistant, antibiotic-treated, and insecticide-susceptible German cockroaches^23^.

Kakumanu et al.^22^ reported on the microbiota from whole body, whole guts and feces of German cockroaches. The overall mean microbial compositions of all the replicates of lab-reared and field-collected cockroaches were remarkably similar at the phylum level, dominated by *Bacteroidetes,* *Firmicutes*, and *Proteobacteria*^22^. However, Kakumanu et al*.* also observed considerable variation in microbial compositions between samples at different locations, as well as differences among individual cockroaches of opposite sexes from the same location^22^.

The prior research noted above corroborates our findings that the oral administration of an antibiotic effectively reduces bacterial species diversity in German cockroaches. Additionally, these researchers found that relative abundances of bacterial taxa in the gut can vary drastically from individual to individual, location to location, and even among individuals in a laboratory environment kept under different dietary regimes^22,23,49^. We used the same primers to amplify the V4 region as Kakumanu et al*.*^22^ and observed some of the same families. While previous literature supports many of our observations, especially in terms of species observed at the phylum level, there is not complete agreement. For instance, Pérez-Cobas et al*.*^40^ used pyrosequencing to sequence the V1–V3 region instead of MiSeq to sequence the V4 region, as Illumina’s platforms were not as frequently used during the time of publication, although both studies produced similar results in terms of species abundance. Additionally, a different antibiotic was used (doxycycline) and species observed differ slightly when comparing Pietri et al.'s^23^ DE (Destin, FL—Resistant) and ORL (Orlando, FL—Susceptible) to our Dan (Danville, IL—Resistant) and J-wax (Susceptible) strains. Unfortunately, there is also no information on how gut microbiota shift once the ORL—susceptible cockroaches had been fed antibiotics. The largest limitation of our current research is that many of our reads yielded undescribed species, which reduces our ability to compare our research with past studies and sequences.

## Conclusions

Information obtained from sequencing German cockroach gut microbiota can be used to develop specialized microbial control strategies for German cockroaches and potentially other insects. By exposing vulnerabilities in the gut microbiome, researchers can develop products that attack beneficial microbes or augment gut diversity in a deleterious manner. Alternatively, combining an antibiotic—or perhaps another antimicrobial agent—with an active ingredient in a pesticide formulation may have unintended consequences. Ramifications include (but are not limited to) gut bacterial antibiotic tolerance, decreased insecticide efficacy through reduced bioactivation of pro-insecticidal compounds, or overall reduction in bait consumption due to dysbiosis. All of these possibilities should be important considerations when developing pesticides that act through microbial inhibition.

Contrary to our original hypothesis, the gut microbiomes of Danville (R) and J-wax (S) German cockroaches are not significantly different on their own, but the introduction of orally ingested kanamycin eliminated certain taxa while increasing the relative abundance of others. This shift and apparent dysbiosis revealed important cockroach strain differences which may extend to the host population level. *Stenotrophomonas* spp. can colonize a gut microbiome with limited other symbionts in the presence of kanamycin. The antibiotic-induced dysbiosis and insecticide tolerance that occurred in the resistant strain suggest new, exciting mutualistic relationships between gut microbiota and their insect hosts. These microbes may have a role in modulating insecticide toxicity or changing feeding behavior, whether to the benefit or detriment of the host. The mechanisms of antibiotic resistance, as well as potential insecticide degradation and metabolism should be investigated further in *Stenotrophomonas*. More research is needed to determine the specific phylogenetic classifications of many undescribed species discovered in the experiment, as well as their functions, structures, and relationships to the German cockroach host. Once these relationships have been explored more extensively, researchers will have a better understanding of how to develop products aimed at controlling German cockroach by engineering dysbiosis or by building stronger levels of insecticide selectivity and safety. The research presented here is an important initial step towards developing more effective products that can better manage this important public health pest.

## Materials and methods

### Insects

Both insecticide-resistant and insecticide-susceptible strains of male German cockroaches were obtained and tested for their ability to resist and detoxify insecticides. The insecticide-resistant strain of *B. germanica* was originally obtained from Danville, IL (Danville-R) and has shown field resistance to Indoxacarb, Abamectin and Fipronil^9^. The insecticide-susceptible strain known as S.C. Johnson Wax susceptible (J-wax-S) is a standard susceptible lab strain that has been in culture for over 70 years with no previous exposure to Abamectin, Fipronil or any other insecticides^9^.

### Rearing and preparation of traditionally raised insects

Methods for rearing were obtained from Gondhalekar and Scharf^52^. Rearing was conducted in 3.8 L plastic containers which were held in a reach-in environmental chamber at 25 ± 1 °C temperature and 12:12 h light:dark photoperiod. The inner top portions of the rearing units were lightly coated with a mixture of petroleum jelly and mineral oil (2:3) to prevent the cockroaches from escaping. Each rearing unit contained corrugated cardboard harborages, a water source, and rodent diet (No. 8604; Harlan Teklad, Madison, WI).

### Treatment and subsequent gut extractions

Adult male cockroaches were separated into four treatment groups: Danville (insecticide-resistant) roaches treated with/without antibiotics and J-wax (insecticide-susceptible) roaches treated with/without antibiotics (Table 2). Treatments were held in groups of ten male adult cockroaches per petri dish (each dish containing a single pellet (approx. 1 g) of Purina kitten chow (number 100137; Nestlé Purina, Neenah, WI) along with 1.5 mL of either NanoPure water or kanamycin-infused NanoPure water) for 72 h before the gut extraction was conducted. Kanamycin sulfate (CAS 25389-94-0; Acros Organics/Thermo Fisher Scientific, Fair Lawn, NJ) was dissolved in 1.5 mL NanoPure water at 50.0 µg/mL (5% w/v). This concentration was chosen as it was determined to be the highest concentration of kanamycin that could be fed to the cockroaches over 72 h without causing mortality higher than the control treatment. The control group received only 1.5 mL NanoPure water. The whole gut, including the bacteria inside of the gut, of these cockroaches was extracted and homogenized in PBS (Phosphate Buffered Saline). DNA was isolated from the homogenization of the guts using a BDC 2010 homogenizer at 70 rpm (Caframo, Georgian Bluffs, ON, Canada) (10 ups and downs).

**Table 2:** Summary of 12 experimental groups sequenced.

Dan-Ctrl (5 guts)	Dan-Kan (5 guts)	J-wax-Ctrl (5 guts)	J-wax-Kan (5 guts)
Dan-Ctrl (5 guts)	Dan-Kan (5 guts)	J-wax-Ctrl (5 guts)	J-wax-Kan (5 guts)
Dan-Ctrl (5 guts)	Dan-Kan (5 guts)	J-wax-Ctrl (5 guts)	J-wax-Kan (5 guts)

Groups are categorized by strain and treatment, with three replications per strain and treatment combination (sample size = 5 guts).

### Insecticide bioassays

Kanamycin was the antibiotic used in the main experiment, as it is a broad-spectrum antibiotic shown to reduce the microbial community inside insect guts^53^. Kanamycin was applied at 50.0 µg/mL (5% w/v) and dissolved in NanoPure water. Cockroaches did not receive food or water for 24 h prior to exposure to the food pellet (consists of a kitten diet pellet plus insecticide diluted in acetone). Roaches were held with food pellet for 72 h before final mortality was assessed. Treatments were evaluated for average percentage mortality every 24 h until the 72-h holding period is complete—the 72-h mortality score is used when calculating the LC50 measurement for data analysis. An additional experiment was conducted with the same bioassay setup (with no insecticide on the food pellet—only an acetone blank) to control for how much food and water were consumed once each strain was treated with kanamycin. Food and water were measured at both the beginning and end (72 h) of the feeding bioassay.

Insecticides were purchased either from Sigma-Aldrich (St. Louis, MO) or from Thermo Fisher Scientific (Waltham, MA). Insecticides for the bioassay were chosen based on resistance assays performed in Fardisi et al*.*^9^ and were serially diluted in twofold steps with acetone. Treatments contained ten roaches per replicate and were categorized based on insecticide resistance capability, insecticide type, and applied insecticide concentration. A series of 8–9 serial dilutions plus acetone controls were prepared. Abamectin serial dilutions ranged from 25.6 to 0.2 µg/per food pellet, whereas Fipronil serial dilutions ranged from 0.32 to 0.0025 µg/food pellet. Different concentration ranges were tested under different experimental conditions as follows

- Danville + Abamectin [25.6, 12.8, 6.4, 3.2, 1.6, 0.8, 0.4, 0.2 µg/per food pellet],
- Danville + Abamectin + Kanamycin [25.6, 12.8, 6.4, 3.2, 1.6, 0.8, 0.4, 0.2 µg/per food pellet],
- J-wax + Abamectin [25.6, 12.8, 6.4, 3.2, 1.6, 0.8, 0.4, 0.2 µg/per food pellet],
- J-wax + Abamectin + Kanamycin [25.6, 12.8, 6.4, 3.2, 1.6, 0.8, 0.4, 0.2 µg/per food pellet],
- Danville + Fipronil [0.32, 0.16, 0.08, 0.06, 0.04, 0.02, 0.01, 0.005, 0.0025 µg/food pellet],
- Danville + Fipronil + Kanamycin [0.32, 0.16, 0.08, 0.06, 0.04, 0.02, 0.01, 0.005, 0.0025 µg/food pellet],
- J-wax + Fipronil [0.32, 0.16, 0.08, 0.06, 0.04, 0.02, 0.01, 0.005, 0.0025 µg/food pellet],
- J-wax + Fipronil + Kanamycin [0.32, 0.16, 0.08, 0.06, 0.04, 0.02, 0.01, 0.005, 0.0025 µg/food pellet].

Treatments were replicated three times each.

### PCR and sequencing

DNA was isolated from the homogenization of the gut using the QIAGEN DNeasy kit (QIAGEN, Hilden, Germany) and replicated using PCR. For this experiment, incubation time was increased to 16 h (overnight) with a reduced temperature of 37 °C instead of 4 h at 56 °C. This modification increased the quantity of nucleic acids released from gut bacteria which may have been hidden in thick folds of cockroach gut tissue. The gut, including the bacteria inside of the gut, of five roaches of each treatment type were extracted and homogenized in 1.5 mL PBS as detailed above for gut extractions. Bacterial 16S rDNA was PCR-amplified using the previously published primers 338F (ACTCCTACGGGAGGCAGCAG) and 518R (ATTACCGCGGCTGCTGG)^54^. PCR was carried out in a total volume of 15 μL. Each reaction contained 7.5 μL of the Ssofast evagreen supermix reagent (Bio-Rad, Hercules, CA), 0.5 μL of each of the forward and reverse primers (stock 10 μM), 3 ng of template DNA, and nuclease-free water up to 15 μL. The Bio-Rad MyCycler thermocycler reaction conditions were: initial denaturation at 95 °C for 3 min; 30 cycles of denaturation at 95 °C for 15 s, annealing at 55 °C for 15 s, and elongation at 72 °C for 30 s; and a final elongation at 72 °C for 5 min. An additional 5 cycle PCR (with the same conditions) was performed to add barcodes to the resulting 30-cycle PCR product. To avoid PCR bias, the lowest DNA template quantity and the fewest possible PCR amplification cycles were chosen. The integrity and quantity of the amplicons were verified by agarose gel (2%) electrophoresis. DNA concentration was quantified on a nanodrop 2000c spectrophotometer (Thermo Fisher Scientific, Waltham, MA). Samples were sequenced using Illumina Mi-Seq at the Purdue Genomics Core Facility (Purdue University, West Lafayette, IN). The sample pool was titered using a KAPA Library Quantification Kit (Roche, Basel, Switzerland) and run as 5% of a MiSeq 500 cycle kit run (Illumina, San Diego, CA). Each strain of cockroaches (insecticide-resistant and susceptible) and each treatment type (with and without antibiotic treatment) was replicated 3 times, for a total of 12 biological replications each containing 5 whole homogenized guts (Table 1). The results of the sequence will determine the relative abundance of different bacterial taxa between insecticide-resistant and susceptible strains of *B. germanica*.

### Sequence filtering

The sequences were processed using Cui et al.^55^ and Mothur v.1.39.3^56^ following the MiSeq standard operating procedure (SOP) proposed by Kozich et al.^57^. Low-quality sequences were removed from the analysis if they contained ambiguous characters or were over 325 bp. After merging any duplicates, the pre-cluster method was applied to further reduce the sequencing errors produced by the MiSeq Illumina sequencing platform. Chimeras were identified and removed using chimera.vsearch and remove.seqs, respectively. The Silva database (version 138) was used to align and classify the sequences. The sequences were clustered into OTUs at a distance threshold of 0.03 using the average neighbor method. The sequences were sampled to a depth of 24390.

### Statistical analysis

The sequences were subsampled to a depth of 24390 as this was the number of sequences in the sample with the fewest sequences present. Alpha-diversity and species evenness were estimated using the Shannon diversity index and the inverse of Simpson’s evenness index, respectively. All diversity indices were calculated with Mothur v. 1.39.3^56^. The differences in indices among bacteria present in Danville, J-Wax, kanamycin-treated and control samples were analyzed by one-way ANOVA followed by Tukey’s test. NMDS and perMANOVA were performed using the Vegan package in R^58^ to compare and evaluate differences between bacterial communities in the two strains and two treatment types. Barplots of phylum and genera present in each sample were constructed, along with a heatmap containing the 20 most abundant genera in each sample to compare how the bacterial community varies between treatments. *Blattabacterium* were pruned from the downstream analyses as they are present only in cockroach fat bodies and would represent contamination in the context of this sequence.

## Data Availability

The Mothur and R scripts, along with the 16S sequence files are available on GitHub: https://github.com/xcwolfe/German-cockroach-gut-microbiome-toxicity.

## Abbreviations

DAN: Danville, IL Resistant strain
J-WAX: S.C. Johnson Wax Susceptible strain
CTRL: Control treatment
KAN: Kanamycin/kanamycin treatment
OTU: Operational taxonomic unit
