# Author Correction: Differential microbial responses to antibiotic treatments by insecticide-resistant and susceptible cockroach strains (*Blattella germanica* L.)

**DOI:** 10.1038/s41598-021-04470-7

**Published:** 2021-12-30

**Authors:** Zachery M. Wolfe, Michael E. Scharf

**Affiliations:** Department of Entomology, Purdue University, West Lafayette, IN 47907 USA

Correction to: *Scientific Reports* 10.1038/s41598-021-03695-w, published online 17 December 2021

The original version of this Article contained an error in Affiliation 1, which was incorrectly given as ‘Department of Entomology, Purdue University, Tucson, AZ, 47907, USA’. The correct affiliation is listed below:

Department of Entomology, Purdue University, West Lafayette, IN 47907, USA.

The original Article has been corrected.

